# The 26th annual Nucleic Acids Research database issue and Molecular Biology Database Collection

**DOI:** 10.1093/nar/gky1267

**Published:** 2018-12-29

**Authors:** Daniel J Rigden, Xosé M Fernández

**Affiliations:** 1 Institute of Integrative Biology, University of Liverpool, Crown Street, Liverpool L69 7ZB, UK; 2 Institut Curie, 25 rue d’Ulm, 75005 Paris, France

## Abstract

The 2019 Nucleic Acids Research (NAR) Database Issue contains 168 papers spanning molecular biology. Among them, 64 are new and another 92 are updates describing resources that appeared in the Issue previously. The remaining 12 are updates on databases most recently published elsewhere. This Issue contains two Breakthrough articles, on the Virtual Metabolic Human (VMH) database which links human and gut microbiota metabolism with diet and disease, and Vibrism DB, a database of mouse brain anatomy and gene (co-)expression with sophisticated visualization and session sharing. Major returning nucleic acid databases include RNAcentral, miRBase and LncRNA2Target. Protein sequence databases include UniProtKB, InterPro and Pfam, while wwPDB and RCSB cover protein structure. STRING and KEGG update in the section on metabolism and pathways. Microbial genomes are covered by IMG/M and resources for human and model organism genomics include Ensembl, UCSC Genome Browser, GENCODE and Flybase. Genomic variation and disease are well-covered by GWAS Catalog, PopHumanScan, OMIM and COSMIC, CADD being another major newcomer. Major new proteomics resources reporting here include iProX and jPOSTdb. The entire database issue is freely available online on the NAR website (https://academic.oup.com/nar). The NAR online Molecular Biology Database Collection has been updated, reviewing 506 entries, adding 66 new resources and eliminating 147 discontinued URLs, bringing the current total to 1613 databases. It is available at http://www.oxfordjournals.org/nar/database/c.

## NEW AND UPDATED DATABASES

The Nucleic Acids Research (NAR) Database Issue reaches its 26th annual issue in 2019. As ever, the 168 papers within cover the full range of biological research. Among them, entirely new databases account for 64 (Table 1) while 92 cover resources that have previously appeared in the Issue and now return with updates. The remaining 12 papers are updated on databases last published elsewhere (Table 2). The usual categorization is again used: after reports from the major resource collections at the U.S. National Center for Biotechnology Information (NCBI), the European Bioinformatics Institute (EBI) and the BIG Data Center at the Beijing Institute of Genomics, Chinese Academy of Sciences there are these groupings: (i) nucleic acid sequence and structure, transcriptional regulation; (ii) protein sequence and structure; (iii) metabolic and signaling pathways, enzymes and networks; (iv) genomics of viruses, bacteria, protozoa and fungi; (v) genomics of human and model organisms plus comparative genomics; (vi) human genomic variation, diseases and drugs; (vii) plants and (viii) other topics, such as proteomics databases. Many interdisciplinary databases defy easy categorization, encouraging readers to browse the whole issue. The NAR online Molecular Biology Database Collection, classifies databases more finely using 15 categories and 41 subcategories, and can be found at http://www.oxfordjournals.org/nar/database/c.

**Table 1.** Descriptions of new online databases in the 2019 NAR Database issue

Database	URL	Brief description^a^
AleDB	http://aledb.org	Mutations from Adaptive Laboratory Evolution experiments
AlloMAPS	http://allomaps.bii.a-star.edu.sg	Allosteric signaling and mutations in proteins
AmtDB	https://amtdb.org	Ancient mitochondrial DNA
Ancestral Genomes	http://ancestralgenomes.org	Reconstructed ancestral genomes
AWESOME	http://www.awesome-hust.com	Impact of SNPs on post-translational modifications
Bactome	https://bactome.helmholtz-hzi.de	Sequences, transcriptomes and phenotypes of clinical isolates of *Pseudomonas auruginosa*
CAGm	http://www.cagmdb.org	Catalog of all germline microsatellites
CancerSEA	http://biocc.hrbmu.edu.cn/CancerSEA	Cancer single-cell state atlas
CancerSplicingQTL	http://www.cancersplicingqtl-hust.com	Splicing quantitative trait loci in cancer
CellMarker	http://biocc.hrbmu.edu.cn/CellMarker	Cell markers in human and mouse
Cell Model Passports	https://cellmodelpassports.sanger.ac.uk	Human cancer cell models
ChIPprimersDB	http://www.chipprimers.com	qPCR oligonucleotide primers for chromatin immunoprecipitation (ChIP)
CMAUP	http://bidd2.nus.edu.sg/CMAUP	Collective molecular activities of useful plants
CoevDB	http://phylodb.unil.ch/CoevDB	Pairwise nucleotide coevolution
CRISPRlnc	http://crisprlnc.xtbg.ac.cn	Validated CRISPR/Cas9 sgRNAs for model organism lncRNAs
Cucurbit Genomics Database	http://cucurbitgenomics.org	Genomics of the Cucurbitaceae family
dbAMP	http://csb.cse.yzu.edu.tw/dbAMP	Antimicrobial peptide sequences, structures and properties
DSMNC	http://dsmnc.big.ac.cn	Database of somatic mutations in normal cells
EDK	http://bigd.big.ac.cn/edk	Editome disease knowledgebase
EncoMPASS	https://encompass.ninds.nih.gov	Encyclopedia of membrane proteins analyzed by structure and symmetry.
EndoDB	https://vibcancer.be/software-tools/endodb	Gene expression in endothelial cells
ENPD	http://qinlab.sls.cuhk.edu.hk/ENPD	Eukaryotic nucleic acid binding proteins
ETCM	http://www.ehbio.com/ETCM	Encyclopedia of Traditional Chinese Medicine
EVmiRNA	http://bioinfo.life.hust.edu.cn/EVmiRNA	miRNA in extracellular vesicles
EWAS Atlas	http://bigd.big.ac.cn/ewas	Epigenome-Wide Association Studies
EWASdb	http://www.bioapp.org/ewas	Epigenome-Wide Association Studies Atlas
FusionGDB	https://ccsm.uth.edu/FusionGDB	Fusion Gene annotation DataBase
gcMeta	https://gcmeta.wdcm.org	Microbiome research data
Genome Properties	https://www.ebi.ac.uk/interpro/genomeproperties	Pathways and other properties represented by sets of protein families
HACER	http://bioinfo.vanderbilt.edu/AE/HACER	Human ACtive Enhancer to interpret Regulatory variants
HmtVar	https://www.hmtvar.uniba.it	Human mitochondrial DNA variants
iDOG	http://bigd.big.ac.cn/idog	Dog data on genes, SNPs, diseases etc.
iProX	http://www.iprox.org	Proteomics datasets
jPOSTdb	https://jpostdb.org	Japan ProteOme STandard environment
KinaMetrix	http://kinametrix.com	Protein kinase models, conformations and ligands
liqDB	http://bioinfo5.ugr.es/liqdb	Small RNA expression profiles in biofluids
LncBook	http://bigd.big.ac.cn/lncbook	Human lncRNA knowledgebase
MemProtMD	http://memprotmd.bioch.ox.ac.uk	Membrane Proteins Embedded in Lipid Bilayers
MethMotif	http://bioinfo-csi.nus.edu.sg/methmotif	Transcription factor binding motifs coupled with DNA methylation profiles
NucMap	http://bigd.big.ac.cn/NucMap	Nucleosome positioning map across species
OncoBase	http://www.oncobase.biols.ac.cn	Regulatory somatic mutations in human cancers
OpenProt	http://www.openprot.org	Eukaryotic coding potential and proteomes
Pancan-meQTL	http://bioinfo.life.hust.edu.cn/Pancan-meQTL	Methylation quantitative trait loci in cancer
PDX Finder	http://www.pdxfinder.org	Patient-derived xenograft models
PED	http://bigd.big.ac.cn/ped	Plant Editosome Database
PreMedKB	http://fudan-pgx.org/premedkb	Precision medicine knowledgebase
piRTarBase	http://cosbi5.ee.ncku.edu.tw/piRTarBase	piRNA targeting sites
Plasmid Atlas	http://www.patlas.site	Plasmid annotations and metadata
PLSDB	https://ccb-microbe.cs.uni-saarland.de/plsdb	Plasmid annotations and metadata
PopHumanScan	https://pophumanscan.uab.cat	Positively selected regions of the human genome
qPhos	http://qphos.cancerbio.info	Protein phosphorylation dynamics
RetroRules	http://retrorules.org	Reaction rules for synthetic biology
RNact	http://rnact.crg.eu	Experimental and predicted protein–RNA interactions in human and mouse
SAGD	http://bioinfo.life.hust.edu.cn/SAGD	Sex-associated gene database
SEdb	http://www.licpathway.net/sedb	Super-enhancer database
SSRome	http://mggm-lab.easyomics.org	Microsatellites in all organisms
SymMap	http://www.symmap.org	Traditional chinese medicine including symptom mapping
Translocatome	http://translocatome.linkgroup.hu	Translocating human proteins
Trips-Viz	http://trips.ucc.ie	Transcriptome browser
UniLectin	http://www.unilectin.eu	Lectin structure and function
ViBrism DB	https://vibrism.neuroinf.jp	Tomographic transcriptome and co-expression networks in mouse brain
Victors	http://www.phidias.us/victors	Virulence factors
VMH	http://vmh.life	Virtual metabolic human
YeastRGB	http://www.yeastRGB.org	Expression and localization of yeast proteins

^a^For full references to the databases featured in this issue, please see the Table of Contents.

**Table 2.** Updated descriptions of databases most recently published elsewhere

Database	URL	Brief description^a^
CADD	http://cadd.gs.washington.edu	Combined annotation-dependent depletion
GENCODE	http://www.gencodegenes.org	Reference annotation for the human and mouse genomes.
glycosciences.DB	http://www.glycosciences.de/database	Glyco-related databases
Haemopedia	http://haemosphere.org	Haematopoietic expression data
HumanNet	https://www.inetbio.org/humannet	Human gene functional network
LncACTdb	http://www.bio-bigdata.net/LncACTdb	lncRNA–miRNA–gene interactions
MoonDB	http://moondb.hb.univ-amu.fr	Known and predicted multifunctional proteins in model organisms
OrthoInspector	http://lbgi.fr/orthoinspectorv3	Orthologous relations and phylogenetic profiling
piRBase	http://www.regulatoryrna.org	piRNA function and annotation
Stemformatics	http://stemformatics.org	Stem cell and other cell-specific gene expression
UNITE	https://unite.ut.ee	Internal transcribed spacer sequences for fungal identification
Vesiclepedia	http://www.microvesicles.org	Extracellular vesicles

^a^For full references to the databases featured in this issue, please see the Table of Contents.

Among the major global centers, the NCBI (1) reports on new and expanded literature resources, including PubMed Labs (2) a new interface to PubMed, and new sequence database search options. The EBI paper (3) reports on the new databases Single Cell Expression Atlas and PDBe-Knowledgebase. The latter encompasses FunPDBe, an initiative to better harness structural bioinformatics methods and international collaborators to annotate the protein structural data in PDBe. An interesting facility reported by the BIG Data Center paper (4) is their BIG Search which not only scans across the Center’s many resources but accesses indexes from non-Center partner databases on topics as diverse as lncRNAs, plant transcription factors and autophagy-related proteins.

Major returning resources in the ‘Nucleic acid databases′ section include miRBase (5) which focuses on criteria to assess the reliability of microRNA entries and functional annotation from linked target predictions, external manual curation and text mining. For long non-coding RNAs and their targets, LNCipedia (6) contributes an update, also with a major focus on text mining and manual curation. The popular LncRNA2Target database (7) reports a new release with major increases in lncRNAs, targets and lncRNA–target associations. Two papers address piRNAs (the returning piRBase; 8) or their targets (the newcomer piRTarBase; 9). The RNAcentral (10) hub now retrieves ncRNA data from a total of 28 databases. Important progress since the last paper is reported in mapping genome locations, quality control using the Rfam database (11) and functional annotation. Elsewhere two new databases—Plasmid Atlas (12) and PLSDB (13)—allow easy analyses and searches against the ever-increasing number of bacterial plasmid sequences. In resources for transcription factors (TFs), AnimalTFDB (14) now covers 97 animal genomes and includes a variety of new data such as links from TF-SNP pairs to GWAS data, TF gene expression data and protein-protein interaction networks involving TFs. An interesting new database MethMotif (15) integrates TF binding sites with data on DNA methylation, demonstrating the cell type specificity of many TFs in terms of both binding site sequences and methylation profiles.

In the section on protein sequence and structure databases, UniProtKB (16) reports continued exponential growth of much data, growth made manageable by a focus on Reference Proteomes. There is an interesting discussion of the importance of primary manual curation of the Swiss-Prot portion of the database, especially in cases that computational methods would struggle with, and mention of new methods that might contribute to better propagation of that information to the unreviewed UniProtKB/TrEMBL section. Some of these methods use domain assignments from InterPro (17), also contributing an update here describing, among other improvements, annotation with ‘flavors’ of intrinsic disorder and better treatment of discontinuous domains. A contributor to InterPro and major resource in its own right Pfam also has an update paper (18). It reports refinement of many existing entries and the generation of over 800 new protein families using the ECOD structural database (19). This exercise helped refine the description of some Domains of Unknown Function, while the paper also explains how useful annotations for these can flow back from InterPro work to integrate and rationalize content across its multiple contributing databases. For protein structure both the wwPDB consortium (20) and the RCSB (21) report updates, the former pointing out the deposition and validation challenges brought by cryo-EM and serial femtosecond crystallography, the latter listing the impressive variety of external resources integrated into the webpages and describing the incorporation of a new method for description of biological assemblies. Protein post-translational modifications are well-covered by the returning PhosphoSitePlus^®^ database (22), now 15 years old and with exciting new integration of disease-related mutations and protein isoform data, and the iEKPD resource (23) for phosphorylation-related protein domains. They are joined by the new database qPhos (24) covering the dynamics of protein phosphorylation. Among other interesting new arrivals are the Ancestral Genomes database (25), providing reconstructed proteomes for 78 extinct ancestors of current species and two new resources for transmembrane proteins: EncoMPASS (26) focusing on structural similarities and symmetries, and MemProtMD (27) which provides the results of embedding the structures in a lipid bilayer and subsequent coarse-grained dynamics simulations.

A major new arrival in the metabolic and signaling section is the Issue’s first Breakthrough Article. It is increasingly apparent that the gut microbiota and human diet interact in complex ways with human host metabolism to influence health and disease. The Virtual Metabolic Human (VMH) database (28) is an impressively ambitious resource that seeks to capture that complexity through linking together modules containing genes, reactions and chemical compounds within the human cell (both as a whole and in compartments) and gut microbes (more than 600 species). Further resources cover nutrition, both in terms of typical diets and in mapping dietary components onto VMH metabolites. As well as inter-connectedness between these modules, VMH links out to more than 50 other databases. The authors envisage that simulations employing VMH with different diets and different microbial abundances can be used to generate testable hypotheses, for example, regarding correlations between microbiota composition and disease states.

Elsewhere, two returning manually curated databases focus on protein complexes. CORUM (29) covers mammalian complexes and new features include a network-style visualization of subunit interactions and, most interestingly, a recognition and accounting of the impact alternative splicing can have on protein complex function. Complex Portal (30) presents a new interface incorporating visualizations of data from other databases on metabolism, protein structure and gene expression. The very popular database of protein–protein functional associations STRING reports (31) an update to version 11.0. Not only has the number of species covered doubled since the previous version, but the database now allows genome-scale expression dataset uploads and annotation of the resulting networks according to gene-set enrichment analysis. The well-used HumanNet (32), comprising a network of human gene associations with data weighted in a Bayesian framework, reports an update. Already employed data such as protein–protein interactions increased significantly in volume and were supplemented by two novel sources of data, pathway annotations and co-essentiality data. New candidates for involvement in disease can be identified by network-based expansion from a set of known guide genes. Finally, the KEGG database, particularly valued for its pathway reconstructions, reports important new developments in the shape of the KEGG NETWORK and KEGG VARIANT components (33). These human-specific elements allow the integration of variants such as cancer-related mutations of signaling proteins into networks (derived from KEGG’s original pathways) in order to visualize the effects of perturbation on disease-related pathways. A network variation map summarizes the impacts of a set of perturbants—which may also include viral proteins, environmental factors and drugs—on a given pathway.

The microbial genomics section contains updates from twin Joint Genome Institute databases. The IMG/M database of genomes, metagenomes and metatranscriptomes reports growth of around 60% in just 2 years (34). Its interface is improved in a variety of ways, including a powerful new search capability, BLAST searches against specific and bespoke databases, and powerful statistical comparisons of gene function between groups. IMG/M links to genomic metadata in the longstanding GOLD database, also reporting an update here (35). The IMG/VR viral genomics resource has tripled in size in the same period and now includes improved viral host prediction and geographic mapping for uncultivated viral genomes (36). Two virulence factor (VF) databases are included this year. The first, the well-established VFDB (37) reports a new automated tool, VFanalyzer, for the identification of VFs in complete or draft bacterial genomes. It combines sequence similarity searches with a consideration of genomic context to achieve performance that is reportedly comparable with human curation. The new Victors database (38) has manually curated information from over 5000 VFs and a broader scope—encompassing bacterial, viral, parasitic and fungal VFs—than comparable resources.

Human and model organism genomics again has a strong presence, starting with the second of the Issue’s Breakthrough Articles. Vibrism DB (39) contains anatomical, gene expression and co-expression data at different ages of the mouse brain presenting the information in ways including interactive 3D visualization in a browser. More than 170 000 individual expression maps covering coding and non-coding transcripts are included and co-expression can be viewed in anatomical context or in a network format. The database covers many more transcripts than similar resources, requires few brains to profile expression and cleverly allows users to share URLs that encode scene-setting parameters enabling easy sharing of visualizations. Elsewhere in the section, the major resources Ensembl (40) and the UCSC Genome Browser (41) present their usual updates and are joined by GENCODE (42). A new arrival, the Trips-Viz transcriptome browser (43) allows for mapping of Ribo-Seq and mRNA-seq data onto individual transcripts from seven model organisms. Popular model organism databases such as FlyBase (44), ZFIN (45) and PlanMine (46) are joined by the new resource iDOG (47) which contains an impressive variety of data relating to domestic dogs and other canids, and which is designed to appeal to interested lay people as well as expert researchers. The ArrayExpress functional genomics database marks 15 years since its first appearance in this journal with an update (48) reporting the increasing proportion of submissions coming from single cell studies and the challenges of capturing appropriate metadata.

A large number of databases in the areas of human genomic variation, diseases and drugs are included in this Issue. The popular returning database GWAS Catalog (49) now includes studies from over 3500 publications, while a pair of new databases—EWAS Atlas (50) and EWASdb (51)—each service the fast-growing area of Epigenome-Wide Association Studies. The new database PopHumanScan (52) processes population genomics data from last year’s PopHuman database (53) revealing almost 3000 candidate human genome regions under positive selection. They are linked to relevant literature in the database while users are also invited to contribute their own candidate regions for curation. CADD (54) is a major new arrival and is a very popular measure for predicting the deleteriousness of genome variants. Its classification model is trained using a large number of features, including protein, genome and epigenome information. The deleteriousness of both single nucleotide variants and short insertions or deletions can be predicted. A new database, AWESOME (55), specifically addresses the impact of single nucleotide variants on protein post-translational modifications, considering the effects of around 1 million variants from dbSNP (56) on six different modifications. The venerable OMIM database, linking genes and phenotypes such as inherited disorders, reports continued strong growth of around 300 new phenotypes per year such that over 6000 phenotypes are now linked to around 4000 genes (57). Several cancer databases are covered, including the popular curated catalogue of cancer mutations COSMIC (58). The 3D protein structural consequences of cancer-related mutations are covered in COSMIC-3D and in the dedicated resource Cancer3D (59). The roles of long non-coding RNAs in cancer are the subject of the returning database Lnc2Cancer (60), while disease in general is covered by the similarly popular and hugely expanded LncRNADisease (61). *In vivo* study of cancers will be facilitated by the new Cell Model Passports database (62) which contains standardized information for over 1200 cell models enabling a rational choice of cell model for a particular purpose to be made.

Plant databases include the return of the Genome Database for Rosaceae, celebrating 15 years (63), and the new arrival of the Cucurbit Genomics Database (64), covering important crops such as pumpkin, melon and cucumber. Another new arrival, CMAUP (65), aims to catalogue the chemical compounds present in plants of different kinds (edible, medicinal, garden etc.) and distributions, linking them to effects on human proteins, pathways and diseases. It includes over 5000 plants and almost 50 000 ingredients. Two important glycobiology databases are included in the last section. The returning glycosciences.DB (66) reports a new interface and new search options, including the ability to search with glycan (sub-)structures. The newcomer UniLectin3D (67) contains structural information on lectins, their ligands and their interactions. It features a wide range of visualization options and ample links out to related resources. This section also contains two major proteomics databases: iProX (68), a new Chinese contributor to the ProteomeXchange consortium and jPOSTdb (69). Following on from jPOSTrepo, also published in the Database Issue (70), jPOSTdb subjects the original raw data to a standardized protocol. Post-translational modifications and protein isoforms can be visualized with further options allowing differential expression analysis and protein set enrichment with respect to KEGG pathways and Gene Ontology terms. Finally, it is always a pleasure to welcome databases addressing entirely new kinds of data. Such is the case of AleDB (71) which records the results of Adaptive Laboratory Evolution experiments whereby microbes are grown in defined conditions and the mutations responsible for improved phenotypes are tracked.

## NAR ONLINE MOLECULAR BIOLOGY DATABASE COLLECTION

With the 26th release of the NAR online Molecular Biology Database Collection (which is freely available at http://www.oxfordjournals.org/nar/database/c), we feature 66 new resources. We continue to monitor the collection to ensure the information is still relevant and resources are running, contacting authors when repeated downtime is detected. Thanks to this verification process we have updated 506 database entries, removing 147 obsolete or discontinued databases.

We are happy to include new databases in the Collection and we encourage authors of resources published elsewhere to contact us. Such suggestions should be addressed to XMF at xose.m.fernandez@gmail.com and should include database summaries in plain text, organized in accordance with the http://www.oxfordjournals.org/nar/database/summary/1 template.
